# 

**Published:** 1910-02

**Authors:** 


					﻿Sixty-fifth Year.
Vol. LXV.	FEBRUARY, 1910.	No. 7
BUFFALO
MEDICAL JOURNAL
Established 1845 by AUSTIN FLINT M. D.

EDITOR:
WILLIAM WARREN POTTER, M. D. 
ASSISTANT EDITORS:
NELSON W. WILSON, M. D.	JAMES WRIGHT PUTNAM, M. D.
ASSOCIATE EDITORS:
ERNEST WENDE, M. D. JOHN A. RAFTER, M. D.
JOHN PARMENTER, M. D.	JOHN A. MILLER, Ph. D.
MAUD J. FRYE, M. D.
				

